# Data science research in sub-Saharan Africa: Ethical considerations in crowdsourcing for community engagement

**DOI:** 10.17159/sajs.2023/14911

**Published:** 2023-05-30

**Authors:** Suzanne Day, Stuart Rennie

**Affiliations:** 1 Department of Medicine, Division of Infectious Diseases, University of North Carolina at Chapel Hill, Chapel Hill, North Carolina, USA; 2 Department of Social Medicine, University of North Carolina at Chapel Hill, Chapel Hill, North Carolina, USA; 3 UNC Center for Bioethics, University of North Carolina at Chapel Hill, Chapel Hill, North Carolina, USA

**Keywords:** data science, ethics, crowdsourcing, community engagement, sub-Saharan Africa

## Abstract

The growth in data science research in sub-Saharan Africa raises important ethical questions for the collection and use of ‘big data’ in this context, with particularly disparate implications for the most vulnerable and marginalised populations. While enhanced public involvement may be able to mitigate some of these risks, data science presents some unique barriers to community engagement efforts, including limited data literacy, lack of transparency in data collection and use, and little opportunity to ‘opt out’ from participation. The participatory approach of crowdsourcing offers a promising solution to address the critical need for community engagement. Crowdsourcing involves inviting a group to contribute solutions to a problem, and then publicly sharing the results for implementation. By crowdsourcing stakeholder ideas for innovative ways to enhance public involvement in data science research, the Research for Ethical Data Science in Southern Africa (REDSSA) project is leading the efforts to close the community engagement gap. Promising strategies that emerge from these efforts will ultimately help to shape more ethical and equitable data science research in Africa as this field continues to grow.

## Ethical issues in data science in sub-Saharan Africa

Data science interventions developed through the collection and analysis of ‘big data’ have been touted for the possibility to address some of the most pressing social issues facing low- and middle-income countries (LMICs). Big data may enable decision-makers in LMICs to better understand patterns of human migration, track deforestation, estimate poverty among a population, and predict epidemic outbreaks.^1^ In the context of sub-Saharan Africa (SSA), data science research and the collection of big data is an emerging field with the potential for rapid expansion, aided through increased use of digital social networks, availability of Internet access, and mobile smartphone usage.^2^ The rise of data science research could have a number of beneficial applications across SSA nations, such as serving to enhance public health through reporting and containment of disease, establishing early outbreak warning systems, priming healthcare providers for timely response, prompting strategic healthcare planning, and mobilising domestic and international stakeholder support.^3^ While these applications have the potential for positive impact on public health and development, guidance to inform the ethical collection and use of big data has not kept pace with the growth in data science approaches in LMICs.^4^

A data justice perspective provides a potential framework for viewing the ethical concerns of data science in SSA. Data justice is an approach that borrows social justice concepts and applies them to pose ethical questions of rights, fairness and protections in the context of big data collection and use.^5^ From a data justice perspective^6^, there are three conditions to consider in order for data-driven approaches to be ethically sound: non-discrimination (i.e. the ability to challenge biased data and avoid discrimination), engagement in the technology (i.e. the ability to make autonomous decisions about how one’s data are collected, shared and used), and visibility (i.e. the ability to be represented in the data while maintaining privacy protections). When applied to data science in the SSA context, these facets of data justice raise multiple ethical red flags.

First, pertaining to the condition of non-discrimination: it is unclear whether and/or to what extent algorithms in growing use in the SSA context based on the collection of big data are being checked for bias, and what potential harms may result from interventions developed based on biased models. For example, while machine learning predictive models of HIV risk in SSA have the potential to inform testing and other prevention services, predictive models may be biased in terms of which populations are identified as being at elevated HIV risk, which can in turn result in further unintended harms via discrimination and heightened monitoring.^7^ Second, pertaining to the condition of engagement in the technology: there are few opportunities to make autonomous decisions about how one’s data are collected and used, and there are many ways that big data can be used by others for less-than-good intentions, including surveillance for population control and exclusion.^8^ In the SSA context, the growing ability to map human mobility using mobile phone geodata may be misused by governments to predict and prevent population migration in times of crisis.^9^ Third, regarding the condition of visibility: while ideally this condition would see the balance of equal representation with adequate protections, the potential risks associated with the growth of data science are unequally distributed; vulnerable and marginalised populations are at greater risk of being insufficiently represented^10^, as well as at risk of disproportionate government surveillance for criminalisation and control^11^. In the SSA context, marginalised populations may be at particular risk given widespread legislation undermining encryption across SSA countries.^12^

The need for public involvement and community engagement in data science research has been increasingly recognised as essential for mitigating the above-noted risks and improving adherence to data justice principles.^13,14^ However, the topic of data science is not one that lends itself easily to established community engagement approaches that have developed for use in other fields. A lack of data literacy (in terms of understanding what kinds of data are collected, how they are collected, and with what purpose) has resulted in a growing rift between the elite (researchers) who are further ‘in the know’ and a largely unaware (or uninformed) public.^15^ In addition, the terms of participation in data science research do not follow typical research participation processes: one cannot exactly ‘opt out’ of the collection of their data via mobile phone technology, for example, without essentially opting out of dominant forms of social connectivity and economic systems. Good participatory practices for community engagement identify those who are ‘participants’ or potential participants in the research as a key group for engagement^16^; yet in the case of data science research, what choice does one truly have? The data that are collected from communities (some of it personal, even if de-identified) are collected without consent, or via consent processes that do not follow the typical informed consent processes used in other fields of research. While the community-led call-to-action for research to produce ‘nothing about us, without us’ has been essential for shaping engagement processes in other fields^17^, this approach has not similarly been a part of traditional public health surveillance.

Despite these challenges, greater community engagement is urgently needed in research involving big data for the sake of better data science and more equitably beneficial research outcomes. In addition to helping to bridge the information gap between data scientists and the public, community engagement can help data science research to better incorporate the values and interests of the public that are not readily captured in the data.^13^ Narrowing the information gap may also help preserve community trust in research institutions and mitigate misinformation about data science as its activities come to be more widely known. In addition, community engagement may help to address some of the unanticipated negative consequences of data science research and potential vulnerable points that are missed in algorithms by providing greater insights into community members’ perceptions of risks and potential solutions for mitigating them.^18,19^ There is furthermore a need for community engagement efforts that are appropriate and feasible for use within the unique social, cultural, economic, and political contexts of data science research in the SSA context.^20^ While there is limited work being done on community engagement for big data research globally, approaches developed in high-income country contexts may not be easily transferrable into SSA settings, for just as there are unique data justice concerns in SSA, so too are there potentially unique engagement needs.

Herein lies a complex dilemma for data science researchers in SSA seeking to enhance community engagement processes: what would be promising approaches for engaging the community on data science research when it is a topic that is not widely understood, when its processes are largely opaque, when the ‘community’ of affected stakeholders may be millions of people, and when people who are technically participating in the research via the collection of their data have little real choice to ‘opt out’, shape or impact the collection and use of their data? Furthermore, how can we tailor engagement approaches to the unique contexts of data science research in SSA? Finally, how can we ensure that engagement approaches for data science research are developed in ways that would be acceptable and of interest to the communities we seek to involve?

## Stakeholder-driven solutions for community engagement

One promising approach for addressing the above-noted dilemmas may lie in crowdsourcing. Crowdsourcing involves inviting a group of experts and non-experts to contribute creative solutions to a problem, and then sharing the results with the public.^21^ Drawing on the concept of crowd wisdom, crowdsourcing is premised upon the idea that one need not be an ‘expert’ to contribute great ideas; thus, as a methodology for intervention development, crowdsourcing is well positioned to disrupt the elitism that communities may experience as a barrier to engagement in data science research.^22^

Crowdsourcing also serves a dual-purpose approach to problem-solving. It is both a way to gain promising stakeholder-driven ideas for potential implementation, and participating in crowdsourcing also serves as a form of community engagement in spreading awareness about a particular issue, involving communities/relevant stakeholders as key contributors to intervention development, and disseminating potential solutions at the community level.^23–26^ It is an inherently participatory process for intervention development, with solutions emerging through a ‘bottom-up’ community-driven process rather than ‘top-down’ researcher-led designs. Additionally, interventions developed through crowdsourced community ideas have been shown to be effective in addressing community concerns and priorities. Crowdsourcing has been successfully used to develop messaging to encourage community engagement in HIV cure research^27^, to promote HIV testing among at-risk populations^28–30^, and to obtain feedback from community members on clinical trial designs^24^. With demonstrated effectiveness in clinical trials^31^, crowdsourcing approaches have been used extensively by health and scientific research organisations as an innovative approach to problem solving, including the US National Academies of Sciences, Engineering and Medicine^32^, the US National Institutes of Health Research Office of Behavioral and Social Science Research^33^, and *The Lancet* Healthy Cities Commission^34^.

It may not be possible to crowdsource ideas that could solve all the dilemmas involved in data science research. For example, while members of the public could participate in crowdsourcing ideas to change how health surveillance data are collected and used, it is unlikely that such solutions would be implementable without being accompanied by substantial changes in the regulatory sphere. Furthermore, with community engagement for data science still in its infancy in SSA, it is unlikely that community members have sufficient understanding of how health surveillance data are currently collected and used to be able to consider how these processes may be intervened upon in ways more aligned with a data justice approach. Improving the baseline understanding of the wider public on the topic of data science could potentially help to improve the ability of lay communities to engage in crowdsourcing initiatives on this topic. For example, one strategy being examined by the REDSSA team is providing patients with infographics and pamphlets explaining how health data are collected and used for data science purposes. Efforts to make the topic of data science more broadly understood are essential for boosting participation in crowdsourcing efforts, and subsequently the quality of crowdsourced solutions; low participation in crowdsourcing runs the risk of producing designs based on only a small fraction of the potential pool of stakeholders, calling into question the extent to which the crowdsourced product reflects community concerns.^35^

In contrast, crowdsourcing ideas for how to improve community engagement in data science research is a more promising possibility – one which avoids the need for in-depth understanding of data science. By instead asking stakeholders to contribute creative ideas for community engagement about data science, drawing on their own experiences, values and priorities regarding the collection and use of big data, we can develop engagement strategies that are reflective of and responsive to community concerns.^21^ In addition, community-driven ideas for engagement approaches in data science research may be potentially more effective than top-down designs, and would be grounded in the actual concerns/gaps identified by the people we need to hear from in said engagement processes, i.e. those who can identify vulnerabilities/unintended negative consequences, if offered the opportunity to participate in a meaningful way. In this way, crowdsourced solutions for overcoming the challenges identified with community engagement for data science (e.g. ideas for how to increase data literacy, and strategies to enhance transparency in data collection and use) would be developed by and for those communities most impacted by said challenges.

There are, however, some important caveats and limitations to consider. Crowdsourcing is not invulnerable to similar biases, exclusions and disproportionate negative impact as noted above regarding data science itself. Who we engage with to contribute ideas, and how we engage them, will substantially impact the kinds of ideas that are contributed to a crowdsourcing approach.^36^ In crowdsourcing ideas for how to enhance community engagement in data science research, there is much to consider regarding how ‘even’ the playing field is for participation in crowdsourcing: while not requiring expert insights into how data are collected and used, communities may still find it a challenging topic to consider given that data science is a topic that may feel highly irrelevant to or removed from people’s daily lives given their heretofore lack of inclusion in decision-making processes.^37^ Crowdsourcing community engagement strategies therefore will require careful consideration to ensure that potential participants are sufficiently informed to feel like they can contribute an idea, as well as to feel like their contributions will be meaningful. In addition, crowdsourcing in SSA presents several unique considerations, including language diversity, a highly heterogeneous population spread over vast geographic areas, and the limits of implementing digital strategies in resource-constrained settings. However, successful crowdsourcing projects in diverse LMIC settings provide methodological blueprints for mitigating some of these challenges.^29,30,38,39^

## Engagement for ethical data science research

Crowdsourcing ideas for engagement strategies in data science research would be one small step towards addressing a heretofore overlooked aspect of the field: the lack of meaningful mechanisms for obtaining community input on ethical issues in the collection and use of big data. While crowdsourcing is not the only way to develop engagement strategies and has its own ethical challenges^36^, it nonetheless offers a participatory starting point for developing meaningful engagement processes. Furthermore, while ethical challenges of crowdsourcing are fairly well known and there are emergent best practices to help mitigate them, the ethical issues related to data science as they play out in SSA is an as-yet little explored landscape. Increased social science research, both qualitative and quantitative, is needed to measure current community awareness of ‘big data’ research in SSA, and explore concerns that communities have in relation to its many forms. Engagement strategies are urgently needed now to elucidate these challenges more clearly if we are to have a hope of shaping the growing data science field in ways more aligned with the pillars of data justice. To this end, the REDSSA project is leading the way in crowdsourcing stakeholder-driven solutions to the problem of a lack of community engagement in research using big data.^40^ The results of this study will have immediate practical use as new data science initiatives are being increasingly implemented across SSA.^41^ It is imperative for the ethical conduct of data science in Africa that innovations in community engagement keep pace with ‘big data’ research and its novel applications.

## Significance:

There is an increasing movement to ‘digitise’ health-related data on the African continent, and to improve local health and health systems using cutting-edge data analytics. While these big data initiatives may be beneficial, and engagement is needed to help maintain public trust in data science, the introduction of new digital technologies raises ethical concerns and challenges for engagement. In this Commentary, we focus on the ethics of using crowdsourcing as an approach to increasing community engagement in data science. We map out key areas of ethical concern related to data science and argue that crowdsourcing serves as a promising strategy for identifying ways in which communities can become more engaged in data science initiatives.
